# Immunogenetics of Hashimoto's thyroiditis

**DOI:** 10.1186/1740-2557-2-1

**Published:** 2005-03-11

**Authors:** Dimitry A Chistiakov

**Affiliations:** 1 Laboratory of Aquatic Ecology, Katholieke Universiteit Leuven, Ch. De Beriotstraat 32, B-3000 Leuven, Belgium

## Abstract

Hashimoto's thyroiditis (HT) is an organ-specific T-cell mediated disease. It is a complex disease, with a strong genetic component. To date, significant progress has been made towards the identification and functional characterization of HT susceptibility genes. In this review, we will summarize the recent advances in our understanding of the genetic input to the pathogenesis of HT.

## Introduction

Hashimoto's thyroiditis (HT) is one of the most common human autoimmune diseases responsible for considerable morbidity in women [1]. It is an organ-specific T-cell mediated disease that affects the thyroid, and genetics play a contributory role in its complexity. To date, significant progress has been made in identifying and characterizing those genes involved in the disease. In this review, we will summarize recent advances in our understanding of the genetic contribution to the pathogenesis of HT.

### Epidemiology and clinical features of Hashimoto's thyroiditis

Goitrous autoimmune thyroiditis, or Hashimoto's thyroiditis is a common form of chronic autoimmune thyroid disease (AITD). The disorder affects up to 2% of the general population [2] and is more common in older women and ten times more frequent in women than in men [3]. In the NHALES III study, performed in the USA, the prevalence of subclinical and clinical hypothyroidism was 4.6% and 0.3% respectively [4]. Another US epidemiological study, the Whickham survey, showed the prevalence of spontaneous hypothyroidism to be 1.5% in females and less than 0.1% in males [5]. These prevalence rates are similar to those reported in Japan [6] and Finland [7]. A significant proportion of patients have asymptomatic chronic autoimmune thyroiditis and 8% of woman (10% of woman over 55 years of age) and 3% of men have subclinical hypothyroidism [8]. According the data of the 20-year follow-up to the Whickham survey cohort, the risk of developing overt hypothyroidism is four times higher in women aged between 60 and 70 years than for women between 40 and 50 years of age [1].

Subclinical hypothyroidism is characterized by an increase in serum thyrotropin (TSH) whilst serum levels of thyroxine (T_4_) and triiodothyronine (T_3_) remain normal. The overt disease is defined by the dramatic loss of thyroid follicular cells (thyrocytes), hypothyroidism, goitre, circulating autoantibodies to two primary thyroid-specific antigens, thyroglobulin (Tg), thyroid peroxidase (TPO), and lowered concentrations of serum TSH and T_4 _[9]. Histological and cytological features of HT include a dense thyroidal accumulation of lymphocytes, plasma cells and occasional multinuclear giant cells. The epithelial cells are enlarged, with a distinctive eosinophilic cytoplasm, owing to increased number of mitochondria [10].

HT has been shown to often coexist with other autoimmune diseases such as type 1 diabetes (T1D), celiac disease, rheumatoid arthritis, multiple sclerosis, vitiligo, etc [11-14]. HT can also be expressed as part of an autoimmune polyendocrine syndrome type 2 (APS-2), which is usually defined by the occurrence of two or more of the following: Addison's disease (always present), AITD and/or type 1 diabetes [15], in the same patient.

**Figure 1:**
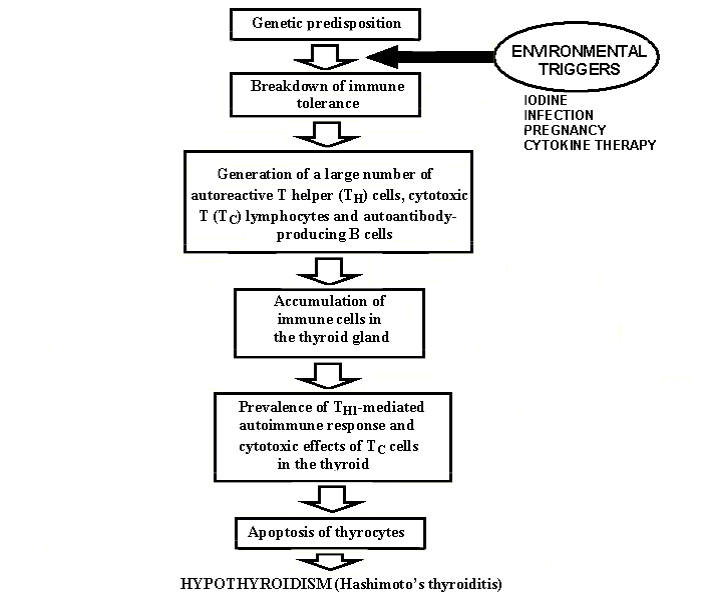
Possible pathogenic mechanism of Hashimoto's thyroiditis. Genetically predisposed individuals could be influenced by an environmental trigger (i.e., dietary iodine, infection, pregnancy, cytokine therapy) that induces an autoimmune response against thyroid-specific antigens by infiltrating immune cells. The autoimmune process results in preferential T helper type 1 (T_H1_)-mediated immune response and induction of apoptosis of thyroid cells that leads to hypothyroidism.

In common with probably all autoimmune disorders, the harmful interaction between internal (genetic) and external (environmental and endogenous) factors is required to initiate Hashimoto's disease (Fig. 1). Environmental triggers of HT include iodine intake [16,17], bacterial and viral infections [18,19], cytokine therapy [20] and probably pregnancy [21,22]. The role of dietary iodine is well defined in epidemiological studies [23,24] and in animal models [25-27] and seems to be the most significant environmental factor to induce thyroiditis.

### Pathogenesis of Hashimoto's thyroiditis

#### Autoimmunity in Hashimoto's thyroiditis

**Figure 2:**
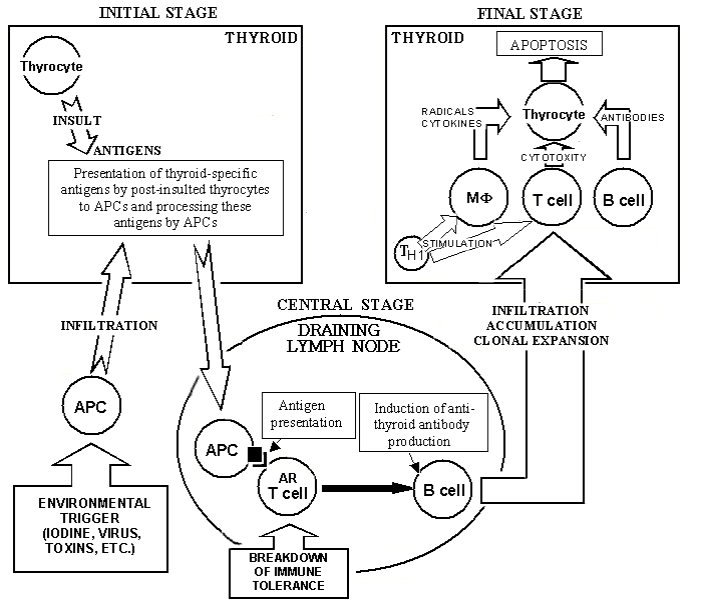
A scheme of autoimmune events in Hashimoto's thyroiditis. In an initial stage, antigen-presenting cells (APC), mostly dendritic cell and macrophage (Mφ) derived, infiltrate the thyroid gland. The infiltration can be induced by an envinromental triggering factor (dietary iodine, toxins, virus infection, etc.) which causes insult of thyrocytes and releasing of thyroid-specific proteins. These proteins serve as a source of self-antigenic peptides that are presented on the cell surface of APC after processing. Taking up relevant autoantigens, APC travel from the thyroid to the draining lymph node. A central phase occurs in the draining lymph node in which interactions between APC, autoreactive (AR) T cells (that survive as result of dysregulation or breakage of immune tolerance) and B cells result in inducing production of thyroid autoantibodies. In the next step, antigen-producing B lymphocytes, cytotoxic T cells and macrophages infiltrate and accumulate in the thyroid through expansion of lymphocyte clones and propagation of lymphoid tissue within the thyroid gland. This process is preferentially mediated by T helper type 1 (T_H1_) cells which secrete regulatory cytokines (interleukin-12, interferon-γ and tumor necrosis factor-α). In a final stage, the generated autoreactive T cells, B cells and antibodies cause massive depletion of thyrocytes via antibody-dependent, cytokine-mediated and apoptotic mechanisms of cytotoxity that leads to hypothyroidism and Hashimoto's disease.

The development of the autoimmune failure of the thyroid is a multistep process, requiring several genetic and environmental abnormalities to converge before full-blown disease develops (Fig. 2). At the onset of disease, major histocompatibility complex (MHC) class II-positive antigen-presenting cells (APC), particularly dendritic cells, and different subclasses of macrophages, accumulate in the thyroid [28,29]. APC present thyroid-specific autoantigens to the naïve T cells, leading to activation and clonal expansion of the latter. Thus, the initial stage of the disease is followed by a clonal expansion phase and maturation of autoreactive T and B lymphocytes in the draining lymph nodes.

In autoimmune thyroditis animal models, genetically determined immune defects have been suggestively linked to the breakdown of immunological self-tolerance that results in the presentation of host autoantigens and expansion of autoreactive lymphocyte clones. These immune defects are associated with the presence of particular MHC class II haplotypes, but other immune and immune regulatory genes (i.e., CTLA-4 and others) are also involved [30-32].

Breakdown of the immune tolerance might occur in several ways including interrupting central tolerance (e.g. deletion of autoreactive T cells in the thymus), defects in maintaining peripheral tolerance (e.g. activation-induced T-cell death and suppressing activity of regulatory T lymphocytes) and anergy (e.g. the expression of MHC class II molecules on non-professional APC). Animal models genetically predisposed to develop an autoimmune disease, and patients with AITD, showed a lack of, or a deficiency in, a subpopulation of regulatory T cells with suppressive function [33-35].

The mechanisms, whereby autoreactive T cells escape deletion and anergy, and become activated, remain uncertain. There is evidence that the thyroid cell itself, by "aberrantly" expressing MHC molecules, can play the role of "non-professional " APS and present disease-initiating antigen directly to the T cells [36,37]. The concept of aberrant MHC class II expression was supported by studies in mice. They developed a type of Graves' Disease (GD) after being injected with fibroblasts coexpressing MHC class II and the TSH receptor (TSHR). TPO antibody production was induced after injection with fibroblasts coexpressing class II molecules and TPO [38,39].

Iodine is a necessary component of normal thyroid hormonogenesis. Incorporation of iodine into thyrosine residues of Tg leads to the formation of mono-iodotyrosine and di-idothyrosine derivates that subsequently undergo an oxidative coupling event resulting in the producing of T_3 _and T_4_. Iodine can promote antithyroid immunity in a number of ways. Several studies suggest that iodination of Tg is crucial for recognition by Tg-reactive T cells [40,41]. Iodine excess can affect the Tg molecule directly, creating new epitopes or exposing "cryptic" epitopes. It has been demonstrated that a highly iodinated thyroglobulin molecule is a better immunogen than Tg of low iodine content [41,42]. Therefore, highly iodinated Tg may facilitate antigen uptake and processing by APC. Additionally, high doses of iodine were shown to directly affect macrophages, dendritic cells, B and T lymphocytes, resulting in stimulation of macrophage myeloperoxidase activity, acceleration of the maturation of dendritic cells, increasing the number of circulating T cells and stimulating B cell immunoglobulin production [25]. Excessive amounts of iodide ion are rapidly oxidized by TPO, thereby generating excessive amounts of reactive intermediates such as hypoiodous acid and oxygen radicals. These oxidative species damage thyrocyte cell membrane by oxidation of membrane lipids and proteins causing thyrocyte necrosis [43]. The state of severe iodine deficiency itself namely leads to a lowering of thyroid autoimmunity and an immunodeficient state in autoimmune-prone BB-DP rats. This hampers the autoreactcive T-cell generation and autoantibody production [25]. A lower degree of Tg iodination also makes this molecule less antigenic [42].

An influx of dendritic cells and macrophages to the thyroid may occur as a consequence of inflammatory events in the gland. Early non-specific necrosis of thyrocytes due to toxins (i.e. iodine, etc.), and perhaps viral or bacterial infection, can attract these cells to the thyroid. Moreover, these immune cells are normal constituents of the thyroid that are able to regulate the growth and function of thyrocytes via interleukin-1 (IL-1) and IL-6-mediated pathways [44].

A central phase of HT is characterized by the recognition of presented autoantigens by the lymphocytes, followed by an apparent uncontrolled production of autoreactive CD4+ T cells, CD8+ cytotoxic T cells and immunoglobulin G (IgG) autoantibodies. Initially, the production of self-reactive cells and autoantibodies occurs in the draining lymph nodes (Fig. 2). Later, the lymphoid tissue often develops directly in the thyroid gland itself. This tissue is generally very well organized, with cords of anti-Tg-antibody-producing plasma cells in the periphery. It is usually non-destructive and shows a peaceful co-existence with adjacent thyrocytes.

Thyroglobulin, the main protein synthesized in the thyroid, serves both in the synthesis and in the storage of thyroid hormones. Human Tg molecules contain at least four thyroid hormone synthesis sites from the iodinated tyrosine residues at positions 5, 2553, 2567 and 2746 [45]. The hormone synthesis sites and the iodine content of Tg play an important role in its autoantigenicity [40]. Tg is one of the major autoantigens in thyroid autoimmunity and serologic studies have shown that there are at least 40 antigenic epitopes on human Tg [16,46]. Tg-antibodies are detected in almost all patients with AITD [47]. Anti-thyroglobulin antibodies were also reported in up to 27% of normal individuals [48]. However, numerous studies have clearly shown that the epitope recognition pattern of the natural anti-Tg antibodies is differented from that of AITD-associated anti-Tg antibodies. Most studies have demonstrarted a restricted epitope recognition pattern of AITD subjects by anti-Tg antibodies, in contrast to polyclonal reactivity observed with anti-Tg antibodies from healthy individuals [49,50]. Human or mouse Tg immunization induces experimental autoimmune thyroiditis (EAT) in mice [51]. The EAT induction is HLA-dependent implying an interaction between the Tg molecule and the MHC glycoproteins [52]. In addition, alterations to Tg could explain interactions between genetic and environmental factors in the aetiology of HT.

Thyroid peroxidase is another significant autoantigen in the thyroid of patients affected with HT and AITD. This enzyme catalyses the oxidation of iodine to an iodinating species that forms iodotyrosines in a Tg molecule and subsequently iodotyronines [53]. TPO antibodies are heterogeneous. To date, around 180 human TPO anribodies have been cloned and sequenced. This allows for the possible identification of major features of the TPO-directed antibodies repertoire during AITD. In Graves' disease patients, heavy chain VH domains of anti-TPO antibodies preferentially use D proximal IGHV1 genes. IGHV3 genes, mainly located in the middle of the immunoglobulin heavy chain gene (IGH) cluster on chromosome 15q11, characterize HT patients more frequently. A large proportion of the anti-TPO heavy chain VH domain comes about following a VDJ recombination process that uses inverted D genes [54,55].

Autoantibodies against other thyroid-specific antigens such as thyrotropin receptor and sodium iodide symporter were also found in serum of HT patients. However, these antibodies occur at low frequency and do not appear to contribute any diagnostic power for HT [56,57].

In a final, destructive step of Hashimoto's thyroiditis, the autoreactive T cells diffusely accumulate in large numbers and infiltrate thyroid parenchyma (Fig. 2). In the BB-DP rat model, T-helper type 1 (T_H1_)-mediated mechanisms involving production of IL-12, tumor necrosis factor-α (TNF-α) and interferon-γ play a major role in the destruction of thyrocytes, rather than T_H2 _type mechanisms directed by IL-4 and IL-10 [58]. The infiltration of activated scavenger macrophages into the thyroid follicles, thus destroying the thyroid cells, is compatible with T_H1_-mediated mechanisms [59]. Fas and Fas ligand (FasL) expression was higher in rats with lympholytic thyroiditis indicating a role of these apoptotic molecules in thyrocyte death [60].

#### Apoptosis in Hashimoto's thyroiditis

Autoimmune responses against specific antigens are primary determinants in thyroid autoimmunity. Other molecular mechanisms including cell apoptosis may play a role in determining the opposite phenotypic outcomes of AITD such as thyroid destruction in HT and thyroid hyperplasia in GD. T-helper lymphocytes produce cytokines that influence both immune and target cells at several levels. The predominance of T_H1 _or T_H2 _cytokines might regulate thyrocyte survival through the induction of pro-apoptotic and anti-apoptotic proteins. T_H1_-mediated mechanisms lead to thyrocyte depletion in Hashimoto's thyroiditis through the involvement of death receptors and cytokine-regulated apoptotic pathways [61,62].

The normal thyroid gland has been shown to act as an immune privileged site having carefully regulated mechanisms of cell death and self-protection against attack by infiltrating activated T-cells induced by apoptosis [63,64]. Cell apoptosis occurs in the normal thyroid at a low level. As new thyrocytes are produced, old cells are destroyed in order to maintain normal thyroid volume and function. Deregulation of apoptosis, which is weakly determined by genetic susceptibility, can lead to destructive processes. Initiation of an out-of-control apoptotic mechanism in thyroid cells may be caused by various non-genetic injuries that affect expression of apoptosis inhibitor molecule Bcl-2 or membrane ligand FasL [65]. Thyrocytes from HT thyroid glands are able to hyperproduce Fas and FasL on their surfaces thus inducing fratricide apoptosis [66]. IL-1β, abundantly produced in HT glands, induces Fas expression in normal thyrocytes, the cross-linking of Fas resulting in massive thyrocyte apoptosis. This can play a role in the progression of Hashimoto's thyroiditis [67].

Immune-mediated apoptosis of thyrocytes is directed by CD8+ cells. Receptors on the target cell are triggered by lymphocyte ligands and/or released soluble factors are delivered to the target cell [68]. Receptors involved in immune-mediated apoptosis include the TNF R1 receptor, the Fas receptor and death receptors DR3 and DR4, whereas soluble mediators include substances such as perforines and TNF [68-70].

The common apoptotic pathway consists of subsequent activation of specific intracellular proteases known as caspases. These caspases are themselves activated by specific proteolytic cleavage or may be activated by cleavage performed by other caspases. The caspase cascade ultimately induces enzymes that progressively destroy the cell and its genetic material, finally lead to cell death. The apoptosis, or programmed cell death, can be initiated by binding death ligands, such as TNF, TNF-related apoptosis-induced ligand (TRAIL) and FasL, to the cell surface. This in turn starts intracellular signal cascading of caspases [71].

Several apoptosis signalling pathways, initiated by molecules such as FasL and TRAIL, have been shown to be active in thyrocytes and may be involved in destructive thyroiditis [72]. Fas-mediated apoptosis seems to be a general mechanism of cell destruction in AITD. In GD patients, reduced levels of Fas/FasL and increased levels of antiapoptotic molecule Bcl-2 favour thyroid cell survival and apoptosis of infiltrating lymphocytes. In contrast, the regulation of Fas/FasL/Bcl-2 expression in HT can promote thyrocyte apoptosis through homophylic Fas-FasL interactions and a gradual reduction in thyrocyte numbers leading to hypothyroidism [61].

Thus, the rate of thyrocyte apoptosis dictates the clinical outcome of thyroid autoimmunity. Though rare in normal thyroid, it markedly increases during HT, but not in GD. Therefore, regulation of thyrocyte survival is a crucial pathogenic determinant.

### Genetics of Hashimoto's thyroiditis

#### Evidence for genetic susceptibility to Hashimoto's thyroiditis

Abundant epidemiologic data (population-based and family-based studies, twin studies) suggest a strong genetic contribution to the development of HT. The disease clusters in families [22,73]. Thyroid abnormalities with clinical outcomes were observed in 33% of offspring of patients with HT or GD [73]. The sibling risk ratio (λ_S_), that is the ratio of the prevalence of disease in siblings to the prevalence in the general population, can be used as a quantitative measure of the genetic contribution to the disease. Usually, a λ_S _of more than five indicate a significant genetic contribution to the disease development. Based on historical data, the λ_S _for AITD is estimated to be greater than 10, supporting a strong case of genetic influence on disease development [74]. Using HT prevalence data from the NHAHES III study, an estimated λ_S _value is about 28 for HT [74].

In Danish twin study, the concordance rates for Hashimoto's disease were 38% for monozygotic (MZ) twins and 0 for dizygotic (DZ) twins [75]. For HT, a recent twin study in California confirmed these results, showing concordance rates of 55% and 0% in MZ and DZ twins, respectively [76]. For thyroid antibodies, the concordance rate in the Danish twin study was twice high in MZ twins (80%) than that in DZ twins [75]. In a recent twin study in the UK, the concordance rates for Tg-antibodies were 59% and 23% in in MZ and DZ twins, respectively [77]. In this study, the concordance rates for TPO-autoantibodies were 47% and 29% in MZ and DZ twins, respectively [77]. These data suggest that HT and other AITD outcomes such as antibody production against thyroid-specific antigens have a substantial inherited susceptibility. HT seems to be a polygenic disease with a complex mode of inheritance. Immunomodulatory genes are expected to play an important role in predisposing and modulating the pathogenesis of Hashimoto's thyroiditis.

#### Animal models of autoimmune thyroiditis

Animal models of AITD still hold immense promise for the discovery of pathways, genes and environmental factors that determine the development of thyroid autoimmunity. Animals affected by experimental autoimmune thyroiditis (EAT) provide a unique opportunity to uncover disease-associated pathways, which are complicated to define in man.

One of the oldest inbred models is the obese strain chicken (OS), which develops goitrous lympholytic thyroiditis with the subsequent atrophic lympholytic thyroiditis followed by a rapid onset of hypothyroidism [78]. The biobreeding diabetes-prone (BB-DP) rat expresses a form of focal lympholytic thyroiditis that under normal conditions does not lead to hypothyroidism [79]. The nonobese diabetes (NOD) mouse strain NOD-H2^h4 ^spontaneously develops iodine-induced autoimmune thyroiditis but not diabetes [26]. In particular, this murine strain has been extensively used to evaluate the role of iodine in the development of autoimmune thyroiditis [16].

EAT can be induced in mice by injecting with murine or human Tg, [80] and in normal syngenic recipients it is induced by the adoptive transfer of *in vitro *activated T cells from Tg-immunized mice [81]. The induced disease is characterized by the production of murine Tg-specific antibodies and infiltration of the thyroid by lymphocytes and other monocytes, with murine or human Tg-specific CD4+ T cells as the primary effector cells [80,82].

Clinical features of EAT induced in the animal models mentioned above are similar to those of human HT. For example, autoimmune thyroiditis in the NOD-H2^h4 ^mouse is induced by dietary iodine that supports epidemiologic data on human populations. In addition, the iodinified mouse represents high levels of IgG2b that is similar to HT patients expressing the predominance of IgG2 subclass, the human analog of murine IgG2b [83]. IgM class generally restricts Tg-antibodies of normal individuals and mice, while HT individuals and affected mice commonly produce Tg-antibodies of the IgG isotype [17]. However, anti-TPO antibodies generally detectable in HT patients could not be found in NOD-H2^h4 ^mice. Despite some differences between EAT and HT, these animal models have greatly contributed to the knowledge concerning the etiology and the pathogenesis of thyroid autoimmunity, most notably on the events occurring in the very early prodromal phases.

Major Histocompatibility Complex (MHC) molecules are thought to play an important role in the initial stages of the development of HT and AITD. MHC molecules, or Human Leukocyte Antigen (HLA) homologs, play a pivotal role in T-cell repertoire selection in the thymus and in antigen presentation in the periphery. Crystal structures of MHC molecules show a peptide-binding cleft containing the variable region of these molecules. Genetic polymorphism of the MHC molecule determines the specificity and affinity of peptide binding and T-cell recognition. Therefore, polymorphisms within MHC class I and class II loci can play a significant role in predisposition to autoimmune disease [84].

A role of selected HLA class II genes susceptible to HT has been significantly clarified using transgenic NOD (H2A^g7^) class II-knockout mice with EAT as a model for HT [85,86]. In mouse genome, the H2 class II locus is homologous to the human HLA class II region [51]. A role for HLA-DRB1 polymorphism as a determining factor in HT-susceptibility, with DR3-directed predisposition and DR2-mediated resistance to the disease, was demonstrated using H2 class II-negative mice injected with HLA-DRA/DRB1*0301 (DR3) and HLA-DRB1*1502 (DR2) transgenes [85]. A role for HLA-DQ polymorphism was shown with human thyroglobulin-induced EAT in HLA-DQ*0301/DQB1*0302 (DQ8), but not HLA-DQ*0103/DQB1*0601 (DQ6), transgenic mice [52]. In summary, DR3 and DQ8 alleles are found to be susceptible, whereas DR2, DR4 and DQ6 alleles are resistant [30,87]. Studies on EAT-developing mice showed the differential effects of class II molecules on EAT induction. Susceptibility can be determined when class II molecules from a single locus, H2A or HLA-DQ, are examined in transgenic mice, but the overall effect may depend upon the presence of both class II molecules H2A and H2E in mice and HLA-DQ and HLA-DR in humans [88]. Polymorphism within DQ alleles can determine predisposition to HT while DRB1 molecules associated with susceptibility to HT may appear to play a permissive role. The combination of susceptibility-inducing HLA-DQ and permissive DR alleles is responsible for the association of the HLA class II region with the disease.

T cells recognize an antigenic peptide via interaction of their membrane T cell receptors (TcR) with antigen-MHC complexes presented on the surface of APC. Biased or restricted TcR gene use has been reported in a variety of human or murine autoimmune diseases [89]. Biased TcR V gene in intrathyroidal T cells was also observed in mice with spontatenous (NOD strain) or human Tg-induced (CBA/J strain) thyroiditis. This confirms the primary role played by T cells in initiating EAT and the phenomenon of oligoclonal expansion of intrathyroidal T lymphocytes in early thyroiditis [90]. Sequencing of amplified TCR V beta cDNA showed that within each NOD thyroid sample at least one of the overexpressed V beta gene families was clonally expanded. For example, in the CBA/J mouse immunized with human Tg, clonally expressed T cells were shown to primarily express the murine TcR Vβ1 and Vβ13 sequences [91].

A new murine model that developed destructive thyroiditis with histological and clinical features comparable with human HT has been recently reported [92]. The transgenic mice express the TcR of the self-reactive T-cell clone derived from a patient with autoimmune thyroiditis. The T-cell clone is specific for the autoantigen thyroid peroxidase (TPO) peptide comprising amino acid residues at positions 535–551 (TPO_535–551_) of the TPO amino acid sequence. This includes a cryptic epitope (TPO_536–547_) preferentially displayed after endogenous processing during inflammation [93]. These results underline the pathogenic role of autoreactive human T cells and the potential significance of recognition of cryptic epitopes in target molecules such as TPO for inducing thyroid-specific autoimmune response.

The two-signal theory for T cell activation requires TcR engagement of its cognate antigen-MHC complex and CD28 binding to B7 ligands (B7-1 and B7-2) on APC. Activation of T cells results in increased expression of the cytotoxic T cell antigen-4 (CTLA-4) molecule that shares homology with CD28. Although B7-1 (CD80) and B7-2 (CD86) expressed on APC can bind to both CD28 and CTLA-4 (CD152), because of higher affinity, they preferentially bind to CTLA-4 on activated T cells and attenuate the T cell response [94].

The importance of CTLA-4 in the down-regulation of T cell responses and in the induction of anergy and tolerance to alloantigens, tumors and pathogens, has been clearly demonstrated in experiments with CTLA-4 deficient mice. The mice developed a severe inflammatory disorder due to up-regulated proliferation of T cells [95,96]. CTLA-4 can down-regulate T cell responses involving binding and sequestering B7 molecules from CD28, therefore preventing CD28-mediated co-stimulation. Another possibility is that CTLA-4 through its intracellular domain could actively transmit a negative signal resulting in down-regulation of activated T cells [97]. The crucial role of CTLA-4 in maintaining self-tolerance breakdown of which leads to the initiatition of a primary autoimmune response has been demonstrated in several murine models of autoimmune diabetes [98] and autoimmune thyroiditis [32].

#### Human Leukocyte Antigen class I and II genes

Genes of the human MHC region are clustered on chromosome 6p21 and encode HLA glycoproteins and a number of additional proteins, which are predominantly related to immune response. The MHC locus itself contains three groups of genes: class I genes encoding HLA antigens A, B and C, class II genes encoding HLA-DR, DP and DQ molecules and class III genes [99].

Previous studies in the early 1980s investigated the HLA locus in relation to the genetics of HT. Associations between HLA and HT have both been analysed by serologic typing of HLA and DNA typing using sequence-specific oligonucleotide probe analysis or restriction fragment length polymorphism. In Asians, HLA class I (A2, B16, B35, B46, B51, B54, C3) and HLA class II (DR2, DR9, DR53, DQ4) genes showed an association with the disease [31,100-105]. In Caucasians, HT is associated with HLA class II genes such as DR3, DR4, DR5, DQA1*0301, DQB1*0201 and DQB1*0301 [106-120] but not with the HLA-DP and HLA class I (HLA-A, HLA-B and HLA-C) genes [113,114,121]. However, some studies could not reveal an association between HLA-DQ and DR genes and Hashimoto thyroiditis [114,122,123]. Reports of disease-associated alleles are not consistent, but associations appear to be strongest with alleles in the HLA-DR and -DQ loci. This has also been suggested by studies in transgenic mouse [30,52,85-87].

Early linkage, non-genome-wide studies of the HLA region have failed to detect linkage between the HLA locus and HT [124-129]. Using dataset of 56 US Caucasian multigenerational families, genome-wide scans has revealed a susceptibility locus AITD-1 located on chromosome 6p [130]. The AITD-1 locus is common for both general forms of thyroid autoimmunity, HT and GD [130]. This locus was replicated in the expanded dataset of 102 US Caucasian families but is distinct from the HLA gene cluster [131]. Whole-genome scans of a large family with members affected with vitiligo and HT mapped a HT susceptibility locus that shared both the MHC region and the non-MHC AITD-1 [132]. However, evidence for linkage between the HLA locus and HT (or autoimmune thyroid disease) has not been confirmed by further whole-genome scans of other affected families [133,134], sibling pairs [135], or within HLA-DR3 positive families [120]. The lack of linkage means, for instance, the DR3 gene did not cause the familial segregation of Hashimoto's disease while a relatively strong and consistent association showed that HLA-DR3 conferred a generalized increased risk of HT in the general population. These data did not support a major role for the HLA region in the susceptibility to HT and may imply that the DR3 gene modulates the effect of other non-HLA susceptibility gene.

However, a linkage between the HLA region and HT was recently shown in the data set of 40 US multiplex families affected with AITD and type 1 diabetes [136]. The linkage to HT was found to be weaker than to diabetes, suggesting that additional, non-HLA loci were contributing to the joint susceptibility to AITD and T1D. Among HLA-DR alleles, HLA-DR3 was detected as the only associated gene for Hashimoto's thyroiditis and diabetes [136]. Indeed, DR3 seems to represent the major HLA allele, which contributes to the shared susceptibility to T1D and AITD. These findings, however, need to be replicated in larger data sets because early family [137,138] and case-control [139,140] studies have not shown the unique role for HLA-DR3 allele in conferring shared susceptibility to T1D and thyroid autoimmunity.

The HLA region has been established to be involved in multiple autoimmune disorders [141]. The mechanisms by which HLA molecules influence the susceptibility to autoimmune disorders become more and more clear. Different HLA alleles could have different affinities to autoantigenic peptides. Therefore, certain alleles can bind the autoantigenic peptide, with the subsequent recognition by T cells that have escaped self-tolerance, whereas others may not [142]. The possibility of certain class II alleles to bind and present thyroid-specific antigens such as TSHR or Tg peptides has been shown *in vitro *[143] and in mice with EAT [144].

Thyroid autoantigens need to occur in the thyroid or its draining lymph nodes in order for them to be presented by HLA molecules. It has been suggested that an aberrant intrathyroidal expression of MHC class II molecules by thyrocytes is necessary to initiate thyroid autoimmunity [145,146]. This hypothesis is supported by detection of the expression of HLA class II molecules by thyroid epithelial cells in HT and GD patients [147,148] and in studies on animal models with experimentally induced thyroid autoimmunity [85,145,149,150]. The aberrant expression of HLA class II antigen by thyrocytes can initiate autoimmune responses through direct thyroid self-antigen presentation or a secondary event following on from cytokine secretion by infiltrated T lymphocytes [148,151].

Genetic contribution of HLA varies depending on the disease. HLA involvement in T1D, rheumatoid arthritis or multiple sclerosis is large and can constitute more than 50% of the genetic risk [84,152]. Contributions of HLA alleles as genetic risk factors to HT are much weaker [118,153]. HLA class I and II genes appear to contribute to the autoimmunity in general but not to organ specificity. Their role in the predisposition to HT is rather non-specific [62,117]. The HLA class I and II genes appear not to be the primary HT genes, and are likely to be modulating genes that increase the risk for AITD contribution by other genes. HLA class III and other non-HLA genes, located in the HLA region, are also critical to the immune response. It is possible that HLA associations as seen in thyroid autoimmunity are due partially to genetic variation in these closely linked immune regulatory genes and their linkage disequilibrium with class I and II genes [154].

#### HLA class III genes and non-HLA genes of the HLA region

The HLA class III region lies between class I and II genes and encodes important immunoregulatory proteins such as cytokines [tumour necrosis factor (TNF), lymphotoxin alpha (LT-α) and beta (LT-β)], complement components (C2, C4, properdin factor B) and heat shock proteins (HSP) [155]. Both TNF and LT-α mediate B-cell proliferation and humoral immune responses [154]. TNF has been found to enhance cellular expression of HLA class I and II antigens, and enhances adhesion and complement regulatory molecules in the thyroid gland of HT patients. Alterations to the above could promote the autoimmune process [156]. However, case-control studies showed no association between polymorphisms within the TNF and LT-α genes and HT in Germans [112], UK Caucasians [118] and Koreans [157].

HSP70 gene cluster consists of three genes encoding HSP70-1, HSP70-2 and HSP-Hom proteins. They are expressed in response to heat shock and a variety of other stress stimuli (e.g. oxidative free radicals, toxic metal ions and metabolic stress). HSPs are also important for antigen processing and presentation [158]. Genetic variations within all three HSP70 genes were tested in British patients with HT and no associations were found [118]. Polymorphisms of complement component-encoding genes have not yet been evaluated in relation to HT. Meanwhile, finding a link between frequency disturbances in BI and C4A allotypes and one of the forms of thyroid autoimmunity, postpartum thyroiditis [159], may be an intriguing future study in HT patients.

Other genes crucial to the immune response, including TAP (transporters associated with antigen processing), LMP (large multifunctional protease), DMA and DMB genes are located within the HLA class II region [155]. Protein products of TAP (TAP1 and TAP2) and LMP2 (LMP2 and LMP7) genes participate in the proteolysis of endogenous cytoplasmic proteins into small fragments and subsequent transportation of these self-peptides from the cytoplasm into the endoplasmic reticulum, the site of HLA class I assembly [160]. To date, one investigation has been concerned with the association between TAP1 and TAP2 genes and Hashimoto's thyroiditis. No significant association was observed in the British population [118]. The genetic role of LMP in HT has not yet been examined. An association between the R60 allele of the LMP2 gene and GD was observed [161]. Additionally, quantitative defects in the amount of transcription products of TAP1, TAP2, LMP2 and LMP7 genes were found in lymphocytes of patients with AITD [160]. These findings suggest that defective transcription of HLA class I-processing genes could contribute to the quantitative defect in cell-surface expression in autoimmune lymphocytes in HT. Further evaluation of the role of such class I-processing genes as TAP and LMP is necessary.

DMA and DMB genes are involved in the assembly of HLA class II peptides. These genes encode subunits of a functional heterodimer that is critical for class II antigen presentation [160,162]. Based on nucleotide variation within exon 3, three rare DMB alleles (DMB*01kv1, DMB*01kv2 and DMB*01kv3) have been detected in Korean HT patients while these DMB variants have not been found in healthy subjects [163]. However, these DMB alleles have not yet been functionally characterized. In summary, there is a significant dearth of information on how HLA class III genes and non-HLA genes, located in the HLA region, contribute to the pathogenesis of HT. Further studies are required to clarify the involvement of these genes in HT susceptibility.

#### CTLA-4 gene

The CTLA-4 gene is the most frequently studied of the immune modulatory genes located outside the HLA region, in relation to the genetics of HT. This gene encodes a costimulatory molecule, which suppresses T-mediated immune response and is crucial in the maintenance of peripheral immunological self-tolerance [164]. An inventory of case-control studies based on the association between three polymorphic markers within the CTLA-4 gene [A49G dimorphism in the leader peptide, C (-318) T substitution in the promoter region and a dinucleotide repeat polymorphism at the 3'-untranslated region (3'-UTR)] and HT is reviewed in [165]. Results of these studies, except for those for the C (-318) T single nucleotide polymorphism, suggest that polymorphisms within the CTLA-4 gene are associated with the development of HT.

Family studies showed linkage between CTLA-4 and GD [166], thyroid antibody production [167] and autoimmune thyroid disease [12,62] but not specifically to HT, probably due to lack of their power [129,130,135]. Classical linkage analysis is suitable for detecting susceptibility loci with major genetic effects. CTLA-4 demonstrates a modest but significant effect in the genetics of HT. To detect a locus with a modest genetic effect, a large number (at least 400) of affected families should be tested [168].

This investigation has recently been performed involving about 600 AITD families and more than 1300 affected patients [169]. The CTLA-4 gene has been found to play a critical role in the pathogenesis of autoimmune diseases such as GD, HT and T1D [62,153,169]. Disease susceptibility was mapped in the 6.1-kb 3' untranslating region of CTLA-4. Allelic variation was correlated to altered mRNA levels of soluble form of CTLA-4 [169]. This alternative splice form of CTLA-4 lacks exon 3 encoding the transmembrane domain but maintains exon 2 encoding the ligand-binding domain [170]. The short form of CTLA-4 can bind CD80/86 and inhibit T-cell proliferation [171]. The soluble CTLA-4 (sCTLA-4) is expressed constitutively by T regulatory cells suppressing the effector T-cell response [172]. Its role in autoimmune disease is not exactly clear, but sCTLA-4 was observed significantly more often in patients with AITD [173] and myasthenia gravis [174] in comparison with non-affected subjects. Patients with AITD and myasthenia gravis had an aberrant expression of the CTLA-4 products, with high levels of sCTLA-4 and low levels of the intracellular form [175]. Soluble CTLA-4 might play an important role in immune regulation by binding with the B7 molecules, thus interfering with the binding of CD28 and/or full-length CTLA-4. Interference of sCTLA-4 with B7/CTLA-4 interactions could block suppressive signals transferred via surface-bound CTLA-4. Therefore, high concentrations of sCTLA-4 in serum might contribute to disease manifestations through interference of sCTLA-4 with B7/CTLA-4 interaction.

It may be that the amino acid change at codon 17 of the signal peptide could alter the function of the signal peptide to direct intracellulat trafficking of CTLA-4. In *in vitro *expreriments, the Ala17 (G49) allele was found to represent a translation product, which was not glycosylated in one of two N-linked glycosylation sites [176]. This aberrantly glycosylated product was shown to be further translocated from the endoplasmic reticulum back to cytoplasm and, probably, to become a target for proteolytic degradation. In addition, the distribution of Ala17 CTLA-4 variant on the surface of COS1 cells is significantly less density than the Thr17 variant of CTLA-4 [176]. These fundings suggest that the Ala17 allele is linked to the inefficient glycosylation of CTLA-4, which subsequently could affect suppressing effects of the CTLA-4 molecule. This could also explain observations showing that the G49 allele of the CTLA-4 signal peptide is associated with accelerated proliferation of T lymphocytes in human subjects homozygous for this allele, and with suppression of the downregulation of T-cell activation in response to IL-2 [177,178].

The codon 17 single nucleotide polymorphism (SNP) is shown to be in tight linkage disequilibrium with another SNP situated at position (-318) of the CTLA-4 promoter region and with the (AT)_n _repeat polymorphism at the 3'-UTR of the CTLA-4 gene [176,179-181]. For the C (-318) T SNP, the protective T (-318) allele demonstrated higher promoter activity than the alternative C allele in a luciferase expression assay [182]. Since the (-318) dimorphism occurs in a potential regulatory region, this suggets that this nucleotide substitution may influence the expression of CTLA-4. However, this possibility remains to be explored.

The (AT)_n _repeat polymorphism at the 3'-UTR of the CTLA-4 gene has been shown to affect the expression of this costimulatory molecule [174]. Adenylate- and uridylate-rich elements (AUREs) presented in the 3'-UTRs can regulate stability of eukaryotic mRNAs, and their presence correlates with rapid RNA turnover and translational and posttranslational control [183,184]. The AT repeats in the 3'-UTR of CTLA-4 might represent a special type of of AUREs. CTLA-4 mRNA with longer (AT)_n _alleles have shorter half-lives and, hence, are more unstable [174,185]. Indeed, the (AT)_n _microsatellite in the 3'-UTR influences the mRNA stability. Additionally, the CTLA-4 AT-repeat polymorphism was recently shown to alter the inhibitory function of CTLA-4. The long AT-repeat allele is associated with reduced control of T-cell proliferation and thus contributes to the pathogenesis of GD [186].

The AT-repeat may also affect splicing of one or more of the alternative CTLA-4 transcripts but this should be clarified. Ueda *et al*. [169] showed that another polymorphism (A6230G, or CT60 SNP) located in the first position of the 3'-UTR correlates with higher expression of a soluble CTLA-4. In this study, the highest power of linkage with GD was found for this SNP and three other SNPs (JO27, JO30 and JO31) within a 6.1-kb segment of the 3'-UTR, but not for the (AT)_n _repeat polymorphism [169]. However, no T-cell function data were presented. Thus, further investigations are necessary to evaluate functional significance of these SNPs. Due to the linkage disequilibrium, it is currently not possible to determine whether one, or both, are of physiological importance. It can not be excluded that allele combination of several closely linked CTLA-4 polymorphisms might form a functionally significant haplotype that is directly involved in the susceptibility to autoimmune disease [187,188].

It should be noted that the genomic region 2q33 linked to autoimmune disease contains cluster of three genes encoding costimulatory molecules CTLA-4, CD28 and inducible costimulator (ICOS) [189]. However, genetic studies showed that the AITD gene in the 2q33 locus is the CTLA-4 gene and not the CD28 or ICOS genes [167,169,181].

The CTLA4 gene should be recognised as the first major known non-HLA locus of human autoimmunity and that its role in the pathogenesis of HT is rather general and non-specific [74,153]. Association of CTLA-4 with the production of thyroid antibodies [167,190], an event that often represents the subclinical stage of AITD [1], can explain non-specific mechanism of CTLA-4-mediated susceptibility to the development of thyroid autoimmunity. The association of the CTLA-4 gene with several autoimmune diseases such as T1D [153,169], Addison's disease [191,192], multiple sclerosis [193,194], myasthenia gravis [175] and all clinical outcomes of AITD [74], can also explain the general contribution of CTLA-4 to autoimmunity. Interestingly, AITD, Addison's disease and autoimmune diabetes frequently coexist in patients with the autoimmune polyendocrine syndrome type II as mentioned above. The above disorders seem to share a genetic background, and CTLA-4 could represent a common susceptibility focus for them [195]

#### Other immune regulatory genes

In initial phases of AITD, oligoclonal expansion of T lymphocytes occurs in the thyroid gland. These T cells are restricted by their T cell receptor V gene use [89,90]. Therefore, the TcR may be considered a likely candidate gene for AITD and HT. Early case-control investigations showed a lack of association between HT and the T-cell receptor-α gene in the US white population [111] but not the T-cell receptor-β gene in the Japanese [102]. Linkage analysis using a US Caucasian AITD family dataset [129] and Tunisian affected pedigree [196] has eliminated the T-cell receptor V alpha and V beta gene complexes, located on 14q11 and 7q35, respectively, as candidate genes for susceptibility to thyroid autoimmunity. Therefore, the TcR genes are not major susceptibility genes for HT and AITD.

Another likely candidate among immune-related genes was the IGH gene because HT individuals commonly produce Tg-autoantibodies restricted by IgG class [50]. Early investigations found an association between IgH Gm allotypes and AITD in the Japanese [197,198]. However, these findings have not been confirmed in Caucasians [129,196].

Cytokines are crucial in the regulation of immune and inflammatory responses. Multiple investigations showed the important role of these regulatory molecules in directing autoimmune and apoptotic pathogenic processes, of particular, in central and late stages of the development of HT [72,80,199]. Therefore, cytokine genes might be good candidates for HT. Intrathyroidal inflammatory cells and thyroid follicular cells produce a variety of cytokines, including interleukin-1α (IL-1α), IL-1β, IL-2, IL-4, IL-6, IL-8, IL-10, IL-12, IL-13, IL-14, tumor necrosis factor-α, and interferon-γ [200]. Hunt *et al*. [201] evaluated 15 polymorphisms within nine cytokine genes for IL-1α, IL-1β, IL-1 receptor antagonist (IL1RN), IL-1 receptor 1, IL-4, IL-4 receptor, IL-6, IL-10, and transforming growth factor-β in British patients with AITD. They only found a significant association for one of those. The T-allele of the IL-4 promoter [T (-590) C] polymorphism was associated with lower risk of GD and AITD but not HT [201]. Blakemore et al. [202] failed to find an association between a polymorphic minisatellite in the IL1RN gene and HT in another group of affected patients from UK. Thus, it may be concluded that these genes are not major susceptibility genes for thyroid autoimmunity but need to be further studied.

The autoimmune regulator (AIRE1) gene is known to contribute to the pathogenesis of autoimmune polyendocrinopathy-candidiasis-ectodermal dystrophy (APECED), a rare monogenic autoimmune disease with endocrine components including T1D, adrenal failure, and thyroid dysfunction, with major autoantibodies directed against adrenal, pancreas, and thyroid tissue [203]. However, studies in UK patients showed no relation between a 13-bp deletion at nucleotide 964 in exon 8 (964del13) of the AIRE1 gene, a common disease-associated marker for APECED in British population, and HT [204].

The vitamin D-mediated endocrine system plays a role in the regulation of calcium homeostasis, cell proliferation and (auto) immunity. 1,25-Dihydroxi-vitamin D_3 _(1,25(OH)_2_D_3_) is the most active natural vitamin D metabolite that effectively prevents the development of autoimmune thyroiditis in an animal model [205] and inhibits HLA class II expression on endocrine cells [206]. C/T polymorphism located at intron 6 of the vitamin D 1α-hydroxylase (CYP1α) gene failed to show association with HT in Germans [207]. Two polymorphic markers within the vitamin D-binding protein gene encoding another member of the vitamin D metabolic pathway also showed no association with HT in the German population [208]. However, among two polymorphic sites tested at the vitamin D receptor (VDR) gene, the Fok I(+) allele of the FokI/restriction fragment length polymorphism was found to be associated with higher risk HT in Japanese females [209]. Meanwhile, the VDR gene remains to be a likely candidate for the common autoimmune susceptibility gene because it has been found to be associated with autoimmune disorders such as GD [210], Addison's disease [211], multiple sclerosis [212] and T1D [213].

Thus, a wide variety of non-HLA immune regulatory genes located outside the HLA region showed no significant linkage or association with HT and AITD except for the CTLA4 gene. However, we still cannot estimate whether or not these genes significantly contribute to HT susceptibility due to a serious shortfall in information about their role in this disorder. It cannot be excluded that other genes in linkage disequilibrium with these genes are the susceptibility genes at these loci.

#### Thyroid-specific genes

Antibodies against thyroid peroxidase are one of the most specific features of HT [214]. Therefore, the TPO gene is expected to be a putative candidate responsible not only for susceptibility to HT but also for specific determination between two common outcomes of AITD, such as HT and GD. Genetic transmission of the recognition by antibody of the TPO immunodominant region and the TPO B domain has been described in families affected with HT [215]. This transmission could be explained by genetic variations within the TPO gene. However, case-control studies showed lack of association between the TPO gene polymorphisms and AITD [113,216]. These data suggest that the thyroid peroxidase gene does not play an important role in predisposition to HT. Subsequent studies are necessary to clarify exactly whether this gene is a true susceptibility gene for AITD.

Within the other thyroid-specific gene, the TSHR gene, the T52P amino acid substitute was examined in US white and Thai populations but no association with HT was found [217,218]. Various genome-wide scans have failed to detect linkage between the thyrotropin receptor gene and HT or AITD [130,133-135,219,220]. However, two microsatellites, an (AT)_n _marker at intron 2 of the TSHR gene and a (CA)_n _marker that was mapped to approximately 600 kb of the TSHR gene, have been shown to be strongly associated with HT in Japanese patients [221,222]. The TSHR gene, therefore, does not seem to be a major susceptibility gene for HT, although a minor role cannot be excluded.

Tg-specific autoantibodies are common in AITD. The thyroglobulin gene makes a significant contribution to HT and AITD. Whole-genome scans in Japanese-affected sibling pairs have detected a HT susceptibility locus on chromosome 8q24, with a maximum linkage to marker D8S272 [135]. This marker is separated by 4.6 megabases (Mb) from the Tg gene. Subsequent studies of the mixed US and European Caucasian family dataset has confirmed the susceptibility locus to be on chromosome 8q24, with the maximum linkage to markers D8S514 and D8S284 [74,131,223]. These markers border a large region of chromosome 8 spanning about 15 Mb. The thyroglobulin gene is located within this region. Moreover, a new microsatellite marker Tgms2 inside intron 27 of the Tg gene showed strong evidence of linkage and association with AITD [223,224]. Two new microsatellites have recently been described in introns 29 and 30 of the thyroglobulin gene that can be useful for further linkage studies in families with autoimmune thyroid diseases [225]. Using a high-density panel of SNPs within the human and murine Tg genes, Ban *et al*. [226] identified a unique SNP haplotype, consisting of an exon 10–12 SNP cluster in both genes and, additionally, exon 33 SNP in the human gene, associated with AITD in humans and with EAT in mice. Taken together, these data strongly suggest that the thyroglobulin gene could represent the susceptibility gene for HT and AITD on 8q24 [74,227] and, therefore, be characterized as the first thyroid-specific susceptibility gene for thyroid autoimmunity [228].

The Tg gene spanning over 300 kilobases long is expected to harbour more than one haplotype block associated with AITD since the length of a linkage disequilibrium block of SNPs is shown to be less than 100 kilobases [229]. It seems that this gene is AITD-specific but is not a HT-specific susceptibility gene. The manner in which the Tg gene can be a predisposition to AITD remains unclear. It could be that amino acid variations within the Tg gene can affect the immunogenicity of Tg. The evidence that iodination of thyroglobulin affects its immunogenicity favours this suggestion [230,231]. However, additional studies are required to evaluate that.

Recent investigation in Tunisians showed significant association of two polymorphic microsatellites (D7S496 and D7S2459) close to the PDS gene (7q31) with GD and HT, and one of them, D7S496, was linked to GD only [232]. The PDS gene encodes a transmembrane protein known as pendrin. Pendrin is a chloride/iodide transporting protein identified in the apical membrane of the thyroid gland [233]. Data of Kacem *et al*. [232] suggest that the PDS gene might be considered a new susceptibility gene to autoimmune thyroid diseases, having a different involvement with different diseases. However, studies in other populations are necessary to support a role for the PDS gene in thyroid autoimmunity and HT.

Finally, a role for other genes specifically expressed in the thyroid gland, has yet to be defined. These genes include those encoding thyrotropin-β, thyroid-specific factor-1, sodium iodide (Na^+^/I) symporter and paired box transcription factor-8, among others. They also need to be evaluated for any putative impact on HT.

#### Apoptotosis-related genes

Two polymorphic sites within the FasL gene were recently tested in HT Caucasian patients from Italy and Germany. No association between these polymorphisms and the disorder was shown [234]. Assuming a lack of association of the naturally occurring FasL gene polymorphisms with multiple other autoimmune diseases tested, we conclude that genetic variation within this gene does not contribute to autoimmunity. Inactivating mutations within the Fas and FasL genes are associated with carcinogenesis [235,236]. This situation is common among apoptotic-related genes encoding caspases, death receptors, decoy receptors and death ligands as well as for genes that encode other types of signalling molecules [237]. However, since apoptotic mechanisms play a critical role in pathogenesis and progression of HT, genes associated with programming cell death should be evaluated whether or not they confer susceptibility to HT.

#### Other genes

Due to the prevalence of thyroid autoimmunity in females, gender-related genes could also be considered as putative candidates for HT susceptibility. Some of these genes, such as the CYP19 gene encoding aromatase that participates in estrogen synthesis, and genes for estrogen receptor-α (ESR1) and -β (ESR2), were examined but showed no linkage with HT [238]. The ESR1 and ESR2 genes demonstrated no association with AITD in the Japanese [239,240]. It seems that the CYP19 and both estrogen receptor genes do not predispose to HT and AITD. Other gender-specific genes could contribute to AITD. A possible involvement of such genes has been shown for GD with the discovery of a susceptible locus on chromosome X [238].

The SEL1L gene, encoding a novel transcription factor, was recently described [241]. The gene is located on chromosome 14q24.3-q31 close to the GD-1 susceptibility locus [128,130,219] and considered a likely candidate for thyroid autoimmunity. However, a case-control study in the Japanese population detected no association of a dinucleotide (CA)_n _repeat polymorphism in the intron 20 of the SEL1L gene with AITD [242]. This gene may be a potentially predisposing gene to T1D because it is specifically expressed in adult pancreas and islets of Langerhans [241]. It lies in the vicinity to IDDM11, a susceptibility locus to this autoimmune disease, on chromosome 14q24.3-q31 [243].

A new zink-finger gene designated ZFAT (a novel zink-finger gene in AITD susceptibility region) has been recently found on chromosome 8q24 [244]. The T allele of the Ex9b-SNP10 dimorphism representing an adenine-to-thymidine substitution within intron 9 of this gene was shown to be associated with high risk for AITD in Japanese patients. Functional studies showed that the Ex9b-SNP10 significantly affects the expression of the small antisense transcipt of ZFAT (SAS-ZFAT) *in vitro *and this expression results in the decreasing expression of the truncated form of ZFAT (TR-ZFAT) [244]. This SNP is located in the 3'-UTR of TR-ZFAT and in the promoter region of SAS-ZFAT. Full-length ZFAT and TR-ZFAT encode a protein with unknown function, which has eighteen and eleven repeats of zink-finger domains, respectively. Both molecular variants of ZFAT are expressed in different tissues including peripheral blood lymphocytes, while SAS-ZFAT is exclusively expressed in peripheral blood B cells and represents a non-coding RNA with putative regulatory function [245]. The disease-associated polymorphism can play a significant role in B cell function by enfluencing the expression level of TR-ZFAT through regulation of transcription of SAS-ZFAT. Interestingly, Shirasawa *et al*. found no association of the thyroglobulin gene with AITD when studying different ethnic groups [244]. These results suggest that the ZFAT gene could implicate the susceptibilty to AITD on chromosome 8q24 but that it needs to be strongly replicated in other populations. Additionally, the ZFAT gene should be functionally studied to clarify whether the ZFAT or thyroglobulin gene are true contributors of genetic susceptibility to AITD and HT on 8q24.

#### Non-defined susceptibility loci for Hashimoto's thyroiditis and autoimmune thyroid disease

At present, the CTLA-4 (chromosome 2q33), thyroglobulin (or ZFAT) (8q24) and likely HLA genes (6p21.3) are the only susceptibility loci for HT and thyroid autoimmunity to be mapped. Two HT-specific susceptibility loci that have been detected in mixed Caucasian families from USA and Europe, HT-1 (13q) near marker D13S173 and HT-2 on chromosome 12q in the vicinity of marker D12S351, are still not defined [130]. HT-2 locus has been subsequently replicated in the extended dataset, with a peak linkage close to marker D12S346, which HT-1 does not have [223]. Possible candidate genes for susceptibility to HT positioned within the HT-2 locus may include the BTG1 and CRADD genes. The BTG1 gene encodes B-cell translocation protein-1, which play an immune regulatory role as a negative regulator of the proliferation of B cells [246]. The GRADD gene encodes CASP-2 and RIPK-domain-containing adaptor with death domain, that represents apoptotic function, inducing cell apoptosis via recruiting caspase 2/ICH1, TNF receptor 1, RIPK-RIP kinase and other proteins [247].

AITD-1 locus located on chromosome 6p is very close yet distinct from the HLA region [120,130]. It has been shown that the AITD-1 is positioned in the same location as susceptibility loci for T1D (locus IDDM15) [248] and systemic lupus erythematosus [249]. This may imply that a general autoimmunity susceptibility gene is located in this region. The AITD-1 locus contains an interesting positional candidate gene such the SOX-4 gene, encoding a transcription factor that modulates differentiation of lymphocytes [250].

A whole-genome scan in Japanese showed evidence for linkage with AITD on chromosome 5q31-q33 [135,251]. The 5q31 locus was replicated by recent genome-wide scan in Caucasian population, the Old Order Amish of Lancaster County, from Pennsylvania [220]. This locus harbours a cluster of cytokine genes and, therefore, several positional candidate genes occur in this region and need to be evaluated.

In the Chinese, a whole-genome screening for AITD susceptibility found two chromosome regions (9q13 and 11q12) linked to AITD [134]. Susceptibility genes have yet to be defined within these regions. However, the 9q13 locus harbours a putative candidate gene such as the ANXA1 gene, whose product annexin A1 prevents the production of inflammatory mediators [252]. The 11q12 locus contains several interesting candidate genes encoding immune modulators (CD5 and CD6) and possible components of antigenic peptide processing (PSMC3) and transport (PTH2).

In a large Tunisian family affected with AITD, a susceptibility locus was mapped on 2p24 [133] This locus harbors two possible candidate genes such as the FKBP1B gene, product of which demonstrates immune modulating activity [253], and the TP53I3 gene encoding p53-inducible protein 3 that is involved in p53-mediated apoptotic pathway [254].

These data suggest that both HT and AITD show genetic heterogeneity in different populations. Susceptibility loci differ in their chromosome location depending on the population being tested. The contributory value of these genes to the disease pathology varies significantly depending on the ethnic background. A gene, that has a major effect on the susceptibility to HT in one population, may contribute weakly in other population. To date, several regions of linkage to HT and AITD have been defined. Further studies are required to find a true susceptibility gene in these genomic regions to reveal the functional significance of disease-associated polymorphisms within these genes.

## Conclusion

AITD can be initiated in individuals genetically predisposed to AITD by non-genetic (environmental) triggers such as dietary iodine, infection, pregnancy, cytokine therapy (Fig. 1). This interaction leads to different clinical phenotypes of thyroid autoimmunity such as Graves' disease, Hashimoto's thyroiditis or production of thyroid antibodies. HT and GD are two distinct but related clinical outcomes of AITD. It seems that both thyroid diseases have common pathogenic mechanisms as their initial steps including breakdown of the immune tolerance and accumulation of T lymphocytes in the thyroid gland.

Sequence variants of CTLA-4, associated with increased levels of the soluble form of this immune costimulator and with stability of CTLA-4 mRNA, could play a crucial role in the earliest stages of AITD (i. e. breakdown of self-tolerance and surviving autoreactive T lymphocytes). This role might be sufficient to regulate subsequent steps in the development of autoimmune responses to the production of thyroid autoantobodies [167].

Environmental factors (particularly, iodine intake and infection) could cause insult of the thyrocyte followed by abnormal expression of MHC class I and class II molecules, as well as changes to genes or gene products (such as MHC class III and costimulatory molecules) needed for the thyrocyte to become an APC [255]. In this stage, a modulating role of sequence variants of HLA class II molecules could become pivotal in binding and presenting thyroid antigenic peptides derived from Tg, TPO and TSHR. Genetic variations in Tg, and probably in TSHR and other thyroid-specific genes, might be responsible for generating an autoimmune response.

In later stages, thyroid autoimmunity could be switched towards GD or HT. GD is characterized by T_H2_-mediated switching of thyroid-infiltrating T cells. These induce the production of stimulating anti-TSHR antibodies by B cells and anti-apoptotic mechanisms that lead to clinical hyperthyroidism. In HT, preferential T_H1 _response initiates apoptosis of thyroid cells and results in clinical hypothyroidism [22].

It is clear that a number of loci and genes determine genetic predisposition to HT, with varying effects. These loci could be unique to HT or general for both HT and GD. Several whole-genome scans showed results suggesting that there is significant shared susceptibility to HT and GD [130,131,134,135]. This is also supported by the frequent coexistance of both diseases in affected families [74,133]. Preliminary data suggest that shared genetic susceptibility involves both immune regulatory (i. e. CTLA-4 and HLA) and thyroid-specific genes (i.e. Tg). These genes are not responsible for the determination of pathogenic mechanisms of thyroid autoimmunity distinct for HT and GD. It remains unclear which susceptibility genes are specifically involved in the HT pathogenesis.

The CD40 gene, an important immune modulator, appears to act as a GD-specific susceptibility gene. The gene is located within the 20q11 locus and shows significant linkage to GD, but not to HT, in UK Caucasians [74,130,131,256,257]. Subsequent analysis found the CD40 gene to be associated with GD [258]. However, this finding needs to be independently confirmed in other population samples.

A probable susceptibility gene that could direct switching towards GD or HT is thought to be located within the 5q31 locus, which is linked to AITD and contains a cytokine gene cluster. Different sets of cytokines are known to regulate switching to T_H1 _or T_H2 _type mechanisms [58]. There may be two susceptibility genes, each of which uniquely contributes to the development of HT- or GD-specific pathogenesis. The IL-4 promoter [T(-590) C] polymorphism also appears to be associated with GD, but not with HT [199]. IL-4 mediates T_H2 _type mechanism, which can lead to hyperthyroidism [259].

Another HT-specific susceptibility gene(s) may be an apoptotic gene. Apoptosis of thyroid follicular cells is the hallmark of HT and might be the primary cause of death of thyrocytes compared to T cell-mediated cytotoxity [69].

Thus, it is necessary to identify additional susceptibility genes and disease-associated polymorphisms in apoptotic genes in AITD- and HT-linked loci by using a fine mapping approach and high-density panels of SNPs. Further functional analysis and search for correlations between genotype and phenotype will help to evaluate the role of these genes in the development of autoimmune thyroid disease. Susceptibility genes interact with thyroid autoimmunity [62,130], and the level of these interactions could affect disease severity and clinical expressions. The molecular mechanisms of these interactions is unknown. However, significant progress has been made in identifying susceptibility genes to HT and AITD along with intriguing findings regarding the functional characterization of disease-associated polymorphisms. These should stimulate further studies towards the in-depth understanding of the mechanisms by which these genes contribute to thyroid autoimmunity.

## Competing interests

The author(s) declare that they have no competing interests.

## Authors' contributions

ABFG
